# Toxicity of a combined therapy using the mTOR-inhibitor everolimus and PRRT with [^177^Lu]Lu-DOTA-TATE in Lewis rats

**DOI:** 10.1186/s13550-020-00628-y

**Published:** 2020-04-25

**Authors:** Johannes Zellmer, Hsi-Yu Yen, Lena Kaiser, Erik Mille, Franz Josef Gildehaus, Guido Böning, Katja Steiger, Marcus Hacker, Peter Bartenstein, Andrei Todica, Alexander R. Haug, Harun Ilhan

**Affiliations:** 1 Department of Nuclear Medicine, University Hospital, Ludwig-Maxilimians-University Munich, Munich, Germany; 2 Department of Pathology, Technical University of Munich, Munich, Germany; 3 German Cancer Consortium (DKTK), German Cancer Research Centre (DKFZ), Heidelberg, Germany; 4 Comparative Experimental Pathology, Technical University of Munich, Munich, Germany; 5 Department of Biomedical Imaging and Image-guided Therapy, Division of Nuclear Medicine, Medical University of Vienna, Vienna, Austria

**Keywords:** [^177^Lu]Lu-DOTA-TATE, PRRT, Everolimus, Scintigraphy, Renal clearance

## Abstract

**Purpose:**

Peptide receptor radionuclide therapy (PRRT) with [^177^Lu]Lu-DOTA^0^,TYR^3^-octreotate ([^177^Lu]Lu-DOTA-TATE) and the mechanistic target of rapamycin (mTOR) inhibitor everolimus are both approved for the treatment of neuroendocrine tumours (NET). However, tumour progression is still frequent, and treatment strategies need further improvement. One possible approach could be to combine different therapy options. In this study, we investigated the toxicity of a combined treatment with everolimus and [^177^Lu]Lu-DOTA-TATE in female Lewis rats.

**Methods:**

Animals received 200 MBq of [^177^Lu]Lu-DOTA-TATE once and/or 5 mg/kg body weight everolimus or placebo weekly for 16 weeks and were divided into four groups (group 1, placebo; group 2, everolimus; group 3, placebo + [^177^Lu]Lu-DOTA-TATE; group 4, everolimus + [^177^Lu]Lu-DOTA-TATE). Blood levels of creatinine and blood urea nitrogen (BUN) were assessed weekly to monitor nephrotoxicity, and a full blood count was performed at the time of euthanasia to monitor myelotoxicity. Additionally, renal function was analysed by sequential [^99m^Tc]Tc-mercaptoacetyltriglycine ([^99m^Tc]Tc-MAG3) scintigraphies. Histopathological examination was performed in all the kidneys using a standardized renal damage score (RDS).

**Results:**

Rats receiving everolimus showed a significantly lower increase in creatinine levels than those receiving placebo. Everolimus therapy reduced white blood count significantly, which was not observed for [^177^Lu]Lu-DOTA-TATE. Functional renal scintigraphies using [^99m^Tc]Tc-MAG3 showed a compromised initial tracer uptake after PRRT and slower but still preserved excretion after everolimus. Histology showed no significant RDS differences between groups.

**Conclusion:**

Renal scintigraphy is a highly sensitive tool for the detection of renal function impairment after a combination of everolimus and PRRT. Additional treatment with everolimus does not increase renal and haematological toxicity of PRRT with [^177^Lu]Lu-DOTA-TATE.

## Introduction

Neuroendocrine tumours (NET) are a relatively rare entity of malignancies with increasing incidence and prevalence during the last decades [1, 2]. Around 20% of patients present with metastatic disease at the time of diagnosis and up to 38% during follow-up [1]. As opposed to localized NET, where surgical resection represents a curative approach, the therapy of advanced, metastatic NET remains challenging, and the median survival is reported to be about 12 months [1].

The novel targeted drugs sunitinib, everolimus and telotristat etiprate, which were highly effective in randomized controlled trials, complement pharmacologic therapeutic options such as chemotherapy and the use of somatostatin analogues [3–5].

Peptide receptor radionuclide therapy (PRRT) with [^90^Y]Y-DOTA^0^,Tyr^3^-octreotide [^90^Y]Y-DOTA-TOC or [^177^Lu]Lu-DOTA^0^,TYR^3^-octreotate ([^177^Lu]Lu-DOTA-TATE) has been successfully performed for almost 30 years. Recently, Strosberg et al. reported significantly longer progression-free survival for patients with advanced, metastatic midgut NETs treated with [^177^Lu]Lu-DOTA-TATE in the randomized, multi-centric phase-III NETTER-1 trial [6], which led to the approval of [^177^Lu]Lu-DOTA-TATE by the FDA and EMA. However, up to now combined therapy algorithms have not been evaluated in larger cohorts. A possible approach could be the administration of two or more different agents simultaneously. Since everolimus is known to increase the radiosensitivity in solid tumours treated with external radiation therapy [7, 8], the effects of PRRT and everolimus might be potentiated. These considerations gave rise to the phase-I NETTLE study exploring the maximum tolerated dose of everolimus in a combined therapy with [^177^Lu]Lu-DOTA-TATE [9]. In a small cohort of patients who received a standard PRRT regime, no severe adverse effects where seen up to a daily administered dose of 7.5 mg everolimus. However, groups examining the effect of such combined therapies showed that the combination is less effective and can even promote metastasis in preclinical models using the tumour cell line CA20948 [10, 11]. Moreover, there are several adverse effects for both therapies such as haemato- and nephrotoxicity, which also have to be taken into consideration. Using PRRT with [^177^Lu]Lu-DOTA-TATE, haematotoxicity is rare, and dose limiting nephrotoxicity can be reduced by co-administration of basic amino acids [6]. Nonetheless, so far the augmentation of these toxicities using [^177^Lu]Lu-DOTA-TATE in combination with everolimus has not been analysed in detail, yet. The aim of this work is to evaluate the toxicity of this combined treatment in a rat model using [^99m^Tc]Tc-mercaptoacetyltriglycine ([^99m^Tc]Tc-MAG3) scintigraphies for the longitudinal evaluation of renal function, laboratory chemical analyses (blood count, creatinine, blood urea nitrogen) to further assess nephro- and haematotoxicity as well as a histopathologic preparation and microscopic analysis of the kidneys to assess morphological damages to this organ.

## Methods

### Animals and experimental design

All animal experiments were conducted in accordance with institutional guidelines and approved by the Administrative Panel on Laboratory Animal Care (Government of Upper Bavaria, Germany). We used non-tumour bearing female Lewis rats (Charles River Laboratories, Sulzfeld, Germany), aged 10 weeks, with a median weight of 207 g, which were fed a standard diet and given free access to water. The body weight of all animals was monitored weekly. Animals were divided into four groups. Group 1 (*n* = 15) received placebo, group 2 (*n* = 17) everolimus (5 mg/kg body weight once weekly), group 3 (*n* = 14) a combination of placebo (once weekly) and [^177^Lu]Lu-DOTA-TATE (single injection at the start of the study, mean 200 MBq, range 191–207 MBq) and group 4 (*n* = 16) a combined treatment with everolimus (5 mg/kg weekly) and a single injection of [^177^Lu]Lu-DOTA-TATE at the start ot the study (mean 200 MBq; range 195–212 MBq). Based on the experience of Pool et al., the administered activity of 200 MBq [^177^Lu]Lu-DOTA-TATE represents a trade-off between low and high dose therapy and a potential curative dose after a single injection [11]. Renal function was monitored weekly (respectively every 14 days after week 8) by determination of creatinine and blood urea nitrogen (BUN) in the blood serum by drawing approximately 0.5 ml blood from a tail vein. At the end of the observation period, blood samples from the heart were collected to assess the full blood count. Furthermore, renal function of the rats was evaluated with serial [^99m^Tc]Tc-MAG3-scintigraphies in the remaining half of rats. A baseline scan was performed in a group of 21 randomly chosen, otherwise untreated rats 1 week before the start of the actual treatment. Control MAG3 scans were performed in all animals in the four groups (group 1: *n* = 7, group 2: *n* = 7, group 3: *n* = 6, group 4: *n* = 8) 1, 6, 11 and 16 weeks after the start of the treatment. Laboratory studies (*n* = 8 in groups 1, 3 and 4; *n* = 10 in group 2) and renal scintigraphies were performed in different animals of the same group. All animals were euthanized 16 weeks after the start of the treatment, and the kidneys were prepared for the histopathological examination. No animal had to be euthanized due to severe toxicity prior to the endpoint of 16 weeks post treatment.

### Laboratory chemical analysis

Creatinine and BUN levels in the serum were quantified to monitor kidney function. A total blood count was performed right before euthanasia of animals at the end of the study. All laboratory analyses were performed according to the manufacturer’s protocols and standardized methods at the Institute of Laboratory Medicine of the Medical Centre of the University of Munich. Blood was not diluted. Serum creatinine and BUN concentrations were measured using an Olympus AU5400 analyser (Beckman-Coulter) using the creatinine reagent OSR6178 and the urea reagent ORS6578. Blood count analysis was performed using an XN-2000 analyser (Sysmex, Kobe, Japan). All analyses were performed according to the manufacturer’s protocols.

### Pharmaceuticals and radiopharmaceuticals

Everolimus (formerly known as RAD001) and placebo were kindly provided by Novartis Pharmaceuticals (Basel, Switzerland). We applied a weekly dose of 5 mg/kg body weight chosen in accordance with previously published data for single agent treatment [12]. The pharmaceuticals were freshly prepared from the pre-concentrate once weekly right before oral gavage. In accordance with the manufacturer’s manual, the everolimus pre-concentrate was diluted with 5% glucose solution to a concentration of 2 mg/ml corresponding to an administered volume of ~ 0.5 ml. Equivalent amounts of pre-concentrate and glucose solution were used for the preparation of the placebo solution. ^99m^Tc-mercaptoacetyltriglycine was purchased from Covidien, Neustadt/Donau, Germany, and prepared according to the manufacturer’s manual. No carrier added ^177^Lu was obtained from Isotope Technologies Garching GmbH (Garching, Germany). DOTA^0^,TYR^3^-octreotate was obtained from ABX advanced biochemical compounds (Dresden, Germany). Radiolabeling was performed using 125 μg DOTA^0^,TYR^3^-octreotate according to a previously described protocol [13]. Quality control was performed using thin layer chromatography and HPLC. Radiochemical yield 99.9% and purity > 99.5% (molar activity GBq/mol). All radiopharmaceuticals were administered via the tail vein (with an administered volume of ~ 0.5 ml).

### Renal scintigraphy

[^99m^Tc]Tc-MAG3-scintigraphy was performed as described in previously published protocols [14–17]. Inhalational anaesthesia with 2.0% of isoflurane in pure oxygen was induced and maintained with a concentration of 1.5%. Rats received a standard dose of [^99m^Tc]Tc-MAG3 (50 MBq) solved in 0.3 ml of sterile saline as a bolus via tail vein. Whole body scintigraphic recordings were initiated at the moment of tracer administration. One head of a triple-headed gamma camera (Philips–former Picker–Prism 3000 XP, Cleveland, USA) equipped with a LEHR collimator was on hand. The dynamic planar acquisitions consisted of 420 frames of 5 s each to a total of 35 min. For the baseline scans, 240 frames (20 min) were acquired to reduce the duration of anaesthesia.

In order to analyse generated data sets, the Hermes Dynamic Study display software V4.0 was used (Hermes Gold V2.10, Hermes Medical Solutions, Stockholm/London). Standardized regions of interest (ROI) was drawn for the whole body, both the kidneys, peri-renal background reference regions, the bladder, blood pool in the heart and the site of injection [15]. Further, dynamic data sets of the ROIs were used to create renograms using Microsoft Excel, which depicted the proportion of the kidney activity corrected for the background regions and the whole-body activity corrected for the injection site. The baseline renograms were extrapolated to 35 min using a monoexponential fit of the excretion phase. Eventually, the parameters ‘time to peak’, ‘peak’, ‘IA10min’ and ‘Delta10min’ were extracted from the renograms.

Furthermore, the fractional uptake rate (FUR) was calculated to assess renal clearance from the renograms [16]. FUR is defined as the fractional uptake of a tracer in the blood by an organ per time unit and can be calculated in the following way: FUR = *P*(0) × (*k*_l_+*k*_r_)/[IA]. *P*(0) was obtained by extrapolating backwards, using a mono-exponential fit of *P*(t). The figures *k*_l_ and *k*_r_ are the slopes of the linear uptake (LU) segment of the Patlak-Rutland (PR) plots for the left and right kidneys [17].

### Histopathological analysis

After the kidneys were fixed in 10% neutral-buffered formalin solution, they were dehydrated under standard conditions and embedded in paraffin. All blocks were cut into 2 μm slices. Selected slices were stained with Periodic acid–Schiff (PAS), adjacent ones with Haematoxylin-Eosin (HE) according to standard protocols. Subsequently, renal damage was classified according to Rolleman’s grading scale using a renal damage score (RDS) ranging from grade 0 (no damage) to grade 4 (severe damage) [18, 19]. Briefly, evaluation criteria included the following:

Grade 1—inflammatory infiltrate in the glomeruli, little dilatation of tubules; no basal membrane thickening or protein cylinders

Grade 2—same criteria as for grade 1, however in addition rough protein staining, more pronounced dilation of tubules, basal membrane thickening and mitotic activity; very little protein cylinders in tubules

Grade 3—same criteria as for grade 2, however additional shrinkage in a small number of glomeruli, smaller vascular lumina flat or lost tubular epithelium, strong tubule dilatation and more pronounced basal membrane thickening; more protein cylinders

Grade 4—same criteria as for grade 3, however increased shrinkage of glomeruli leading to optical emptiness; strongly dilated tubules with massive protein cylinders and signs of peripheral fibrosis

The findings of the histopathological examination were recorded using the Excel sheet.

### Statistical analysis

Data are expressed as the means of the treatment groups and the corresponding 95% confidence intervals. A *p* value of *p* < 0.05 was considered as statistically significant. Normality and homogeneity of variance were tested using the Shapiro-Wilk test and Levene’s test. To adjust for multiple testing, two-way analysis of variance (ANOVA) was carried out for parameters measured only once at the end of the observation period. When normality and/or homogeneity requirements were not met, the Scheirer-Ray-Hare (SRH) test was used, with the administration of everolimus or placebo as one and the treatment with or without [^177^Lu]Lu-DOTA-TATE as the second factor in both cases. For repeatedly measured parameters from the blood sampling, the ANOVA or SRH test was applied for the individual differences between the first and last measurement. By covering all events of a certain parameter (in our case the values of all animals in all our groups rather than only the animals of two specific groups), the validity of the tests used is increased. Moreover, the added value of ANOVA and SRH lies in the evaluation of an over-additive or synergistic effect by analysing the impact of a combination of PRRT and everolimus. Statistical analysis of scintigraphy results was performed after obtaining and averaging baseline parameters. Means at baseline were considered as the population standard. *T*-tests were conducted versus the population standard for the average of each group in the follow-up scintigraphies. Pearson’s chi-squared test was used to test for differences among the ordinally scaled values of the histological grading.

## Results

### Body weight

No animal had to be sacrificed due to weight loss. Groups receiving everolimus showed slower weight gain than the corresponding groups receiving placebo. Table 1 gives an overview of the mean bodyweight at baseline and week 16 and the corresponding differences. ANOVA showed that everolimus was significantly associated with slower weight gain (*p* = 0.009), whereas there was no significant impact for [^177^Lu]Lu-DOTA-TATE (*p* = 0.133) or the combination of everolimus and [^177^Lu]Lu-DOTA-TATE (*p* = 0.809).

**Table 1:** Mean bodyweight at baseline, week 16 and corresponding differences with confidence intervals

Bodyweight (g)	Baseline	Week 16	Difference (%)
Placebo	204.0 ± 7.5	230.2 ± 10.5	12.8 ± 2.7
Everolimus	204.7 ± 5.2	222.9 ± 5.6	9.0 ± 2.9
Placebo + [^177^Lu]Lu-DOTA-TATE	208.4 ± 3.8	230.5 ± 10.1	10.6 ± 4.5
Everolimus + [^177^Lu]Lu-DOTA-TATE	208.4 ± 4.5	221.1 ± 10.8	6.0 ± 4.0

### BUN, creatinine and blood count during follow-up

Tables 2 and 3 show the mean values and confidence intervals at baseline and at week 16 at the end of the study and the means of their individual differences. For the differences in BUN levels, no significant influence of the factors everolimus (*p* = 0.166) and [^177^Lu]Lu-DOTA-TATE (*p* = 0.894) or their interaction (*p* = 0.397) was found. In contrast, the increase in serum creatinine levels was significantly lower in the groups receiving everolimus (*p* = 0.023). No significant differences were found for the factor [^177^Lu]Lu-DOTA-TATE (*p* = 0.185) or the interaction of both factors (*p* = 0.308).

**Table 2:** Mean BUN at baseline, week 16 and corresponding differences with confidence intervals

BUN (mg/dl)	Baseline	Week 16	Difference (%)
Placebo	16.9 ± 2.1	19.4 ± 1.8	19 ± 27
Everolimus	15.7 ± 2.0	18.3 ± 1.2	15 ± 11
Placebo + [^177^Lu]Lu-DOTA-TATE	17.9 ± 1.7	19.1 ± 2.2	7 ± 10
Everolimus + [^177^Lu]Lu-DOTA-TATE	16.9 ± 1.3	19.9 ± 1.1	19 ± 12

**Table 3:** Mean serum creatinine at baseline, week 16 and corresponding differences with confidence intervals

Creatinine (mg/dl)	Baseline	Week 16	Difference (%)
Placebo	0.44 ± 0.05	0.45 ± 0.05	4 ± 13
Everolimus	0.44 ± 0.04	0.42 ± 0.04	− 4 ± 10
Placebo + [^177^Lu]Lu-DOTA-TATE	0.45 ± 0.05	0.58 ± 0.16	26 ± 25
Everolimus + [^177^Lu]Lu-DOTA-TATE	0.48 ± 0.04	0.46 ± 0.05	− 1 ± 16

The results of the total blood count at week 16 are shown in Table 4. The mean values of red blood cell (RBC) count, haemoglobin and haematocrit showed similar trends among the different groups. Everolimus treatment had a significant impact on all three parameters, whereas no significant impact was found for [^177^Lu]Lu-DOTA-TATE. Animals treated with everolimus showed higher RBC counts than those treated with placebo (*p* < 0.001). [^177^Lu]Lu-DOTA-TATE treatment resulted in a non-significant reduction of RBC (*p* = 0.063) compared to animals without PRRT. The increase of reticulocytes rate due to everolimus was not significant (*p* = 0.085), whereas platelet counts were reduced significantly by everolimus (*p* = 0.043) and non-significantly by [^177^Lu]Lu-DOTA-TATE treatment (*p* = 0.577). Two-way ANOVA showed a significant reduction in the number of leucocytes (white blood cells, WBC) in the everolimus group compared to placebo (*p* = 0.029). There was no significant effect for [^177^Lu]Lu-DOTA-TATE (*p* = 0.508). Correspondingly, the impact of everolimus on WBC was significant (*p* = 0.002) both in the single treatment and combination group, whereas therapy with [^177^Lu]Lu-DOTA-TATE had no significant impact (*p* = 0.628). Regarding neutrophil counts, the impact of everolimus was significant (*p* = 0.028) whereas [^177^Lu]Lu-DOTA-TATE had no significant impact (*p* = 0.764). This was also the case in the combination of everolimus and [^177^Lu]Lu-DOTA-TATE (p=0.854). Both factors had no significant impact on monocyte counts. Using ANOVA and SRH, no statistically significant interactions were detected for any of the aforementioned parameters.

**Table 4:** Overview of the haematologic parameters measured in the first part of the study at week 16. The ranges mark the 95% confidence intervals

	Placebo	Everolimus	Placebo + [^177^Lu]Lu-DOTA-TATE	Everolimus + [^177^Lu]Lu-DOTA-TATE
RBC (10^12^/l)	8.33 ± 0.20	9.44 ± 0.45	8.05 ± 0.20	8.84 ± 0.35
Haemoglobin (g/l)	144 ± 3	163 ± 7	138 ± 2	153 ± 4
Haematocrit	0.442 ± 0.007	0.500 ± 0.022	0.431 ± 0.011	0.470 ± 0.016
Reticulocytes (‰)	20.5 ± 3.3	23.6 ± 2.5	21.8 ± 2.2	23.1 ± 3.6
Platelets (10^9^/l)	597 ± 103	544 ± 118	545 ± 111	506 ± 137
WBC (10^9^/l)	4.98 ± 0.86	4.49 ± 0.48	4.94 ± 0.61	4.16 ± 0.63
Neutrophils	0.81 ± 0.35	1.00 ± 0.30	0.71 ± 0.15	0.89 ± 0.13
Monocytes	0.12 ± 0.06	0.13 ± 0.07	0.17 ± 0.09	0.15 ± 0.10
Lymphocytes	4.03 ± 0.65	3.34 ± 0.48	4.03 ± 0.59	3.09 ± 0.57

### Scintigraphy

**Fig. 1:**
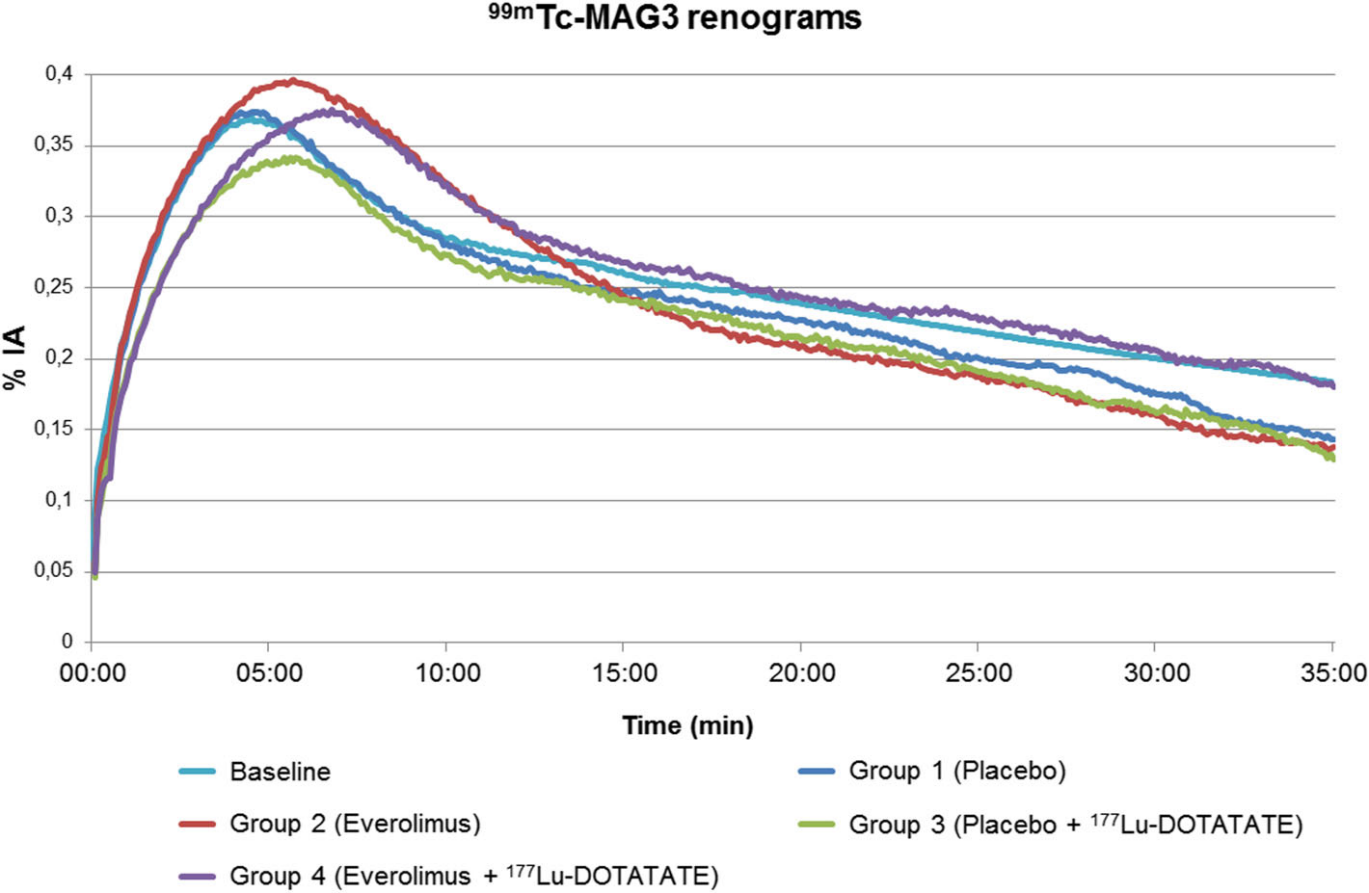
Renograms at follow-up examination 16 weeks after the beginning of each treatment. Reduced steepness of the initial slope reflects impairment of renal function. For clarity, error bars are not shown. The baseline renogram is extrapolated to 35 min using a mono-exponential fit of the excretion phase

**Fig. 2:**
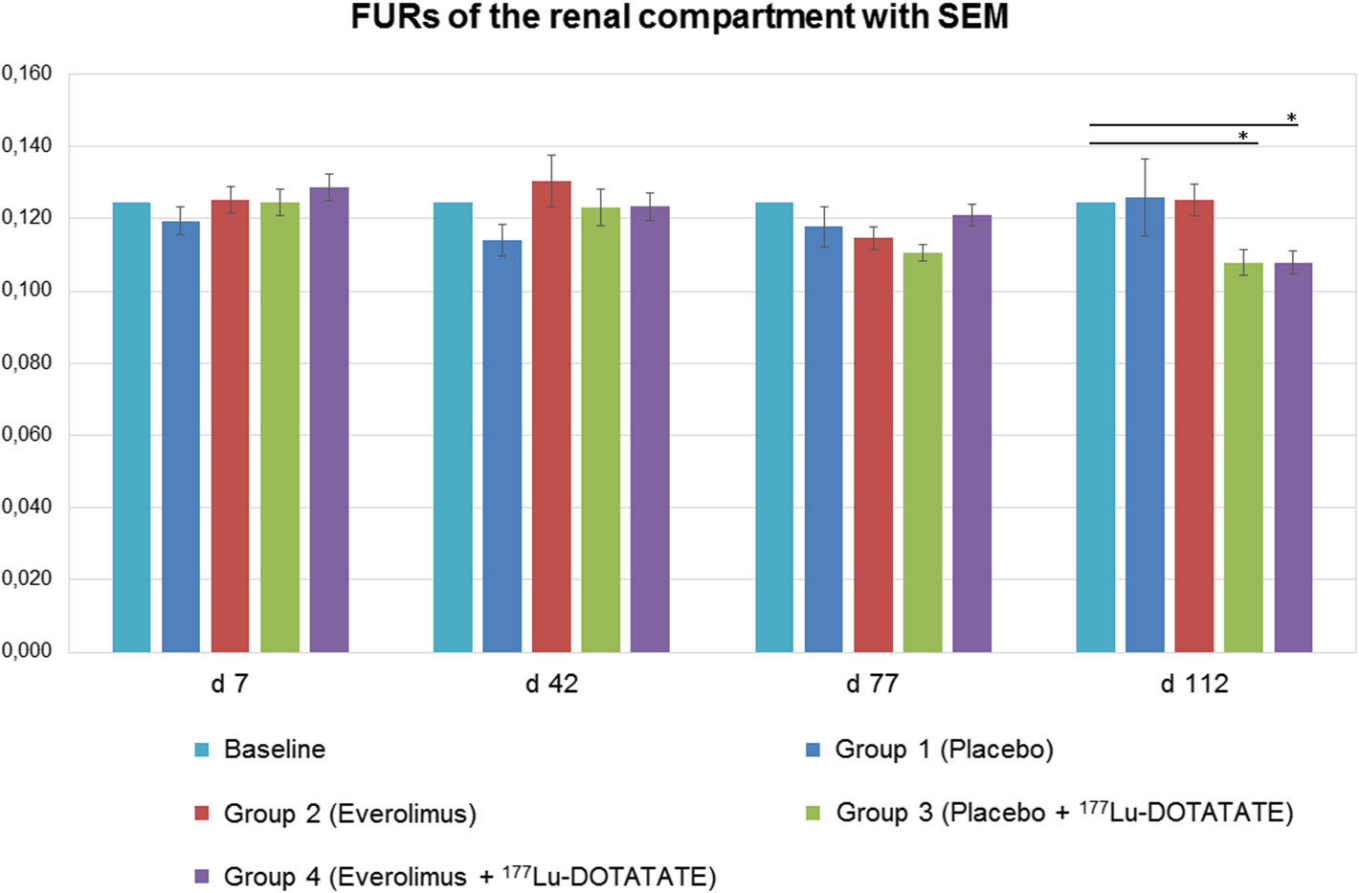
FUR values of the renal compartment at weeks 1 (day 7), 6 (day 42), 11 (day 77) and 16 (day 112) after the start of each treatment compared to baseline values. A significantly decreased FUR was observed in groups 3 and 4 at day 112 compared to baseline values (*p < 0.05). Error bars represent the standard error of the mean

Figure 1 illustrates the renograms in the various groups 16 weeks after start of each treatment compared to baseline values. Results of the scans at week one, six and eleven are not shown. As described previously, preserved renal function is observed by a fast and steep increase of [^99m^Tc]Tc-MAG3 in the kidneys with rapid excretion as well as preserved FUR values comparable to baseline [16, 17]. The renal curve in group 1 (placebo) is almost unchanged compared to baseline. Whereas the initial slope and late excretion in group 2 (everolimus) is also comparable to baseline, the peak is slightly higher (*p* = 0.063) and delayed (*p* = 0.621). The initial slope of both PRRT groups 3 (placebo + [^177^Lu]Lu-DOTA-TATE) and 4 (everolimus + [^177^Lu]Lu-DOTA-TATE) is less steep compared to baseline and to groups 1 and 2. This is reflected by significantly lower FUR values at day 112 (see Fig. 2; *p* = 0.003 for group 3 and *p* = 0.002 for group 4 vs. baseline). Compared to placebo, the administration of everolimus induces a later and higher peak, as already demonstrated between groups 1 and 2. The late excretion appears to be preserved.

### Histopathology

**Fig. 3:**
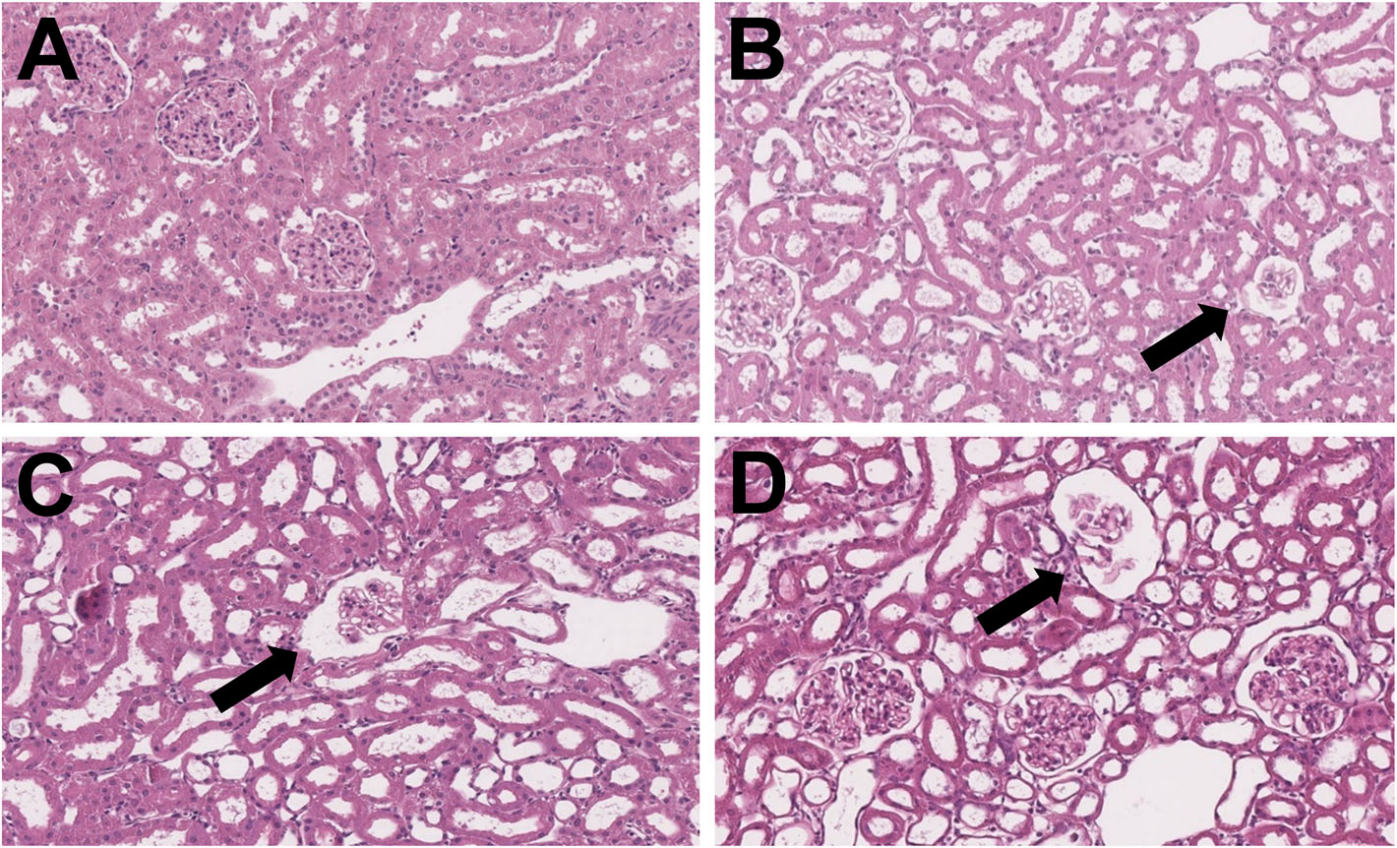
Microscopic images of the kidneys of an animal in group 1 (**a**), group 2 (**b**), group 3 (**c**) and group 4 (**d**). Tubules appear dilated in all animals, and glomeruli shrinkage was found in most animals (black arrows), except for a small number of animals in group 1 (**a**). (HE, magnification × 15)

Figure 3 shows microscopic images of the kidney sections, which are representative for each group (1–4). Each kidney was classified based on the RDS. For the glomeruli, a minimal to slight cell reduction and glomeruli shrinkage was observed in four animals of group 1 and a minimal to moderate in almost all animals of groups 2, 3 and 4. In the tubules, a minimal to marked cell damage, respectively loss of epithelium, was perceived in all animals in all groups. A minimal to marked tubules dilatation was detected in all animals in group 1 and slight to marked tubules dilatation in all animals in groups 2, 3 and 4. A minimal focal inflammation in one animal in group 2 and minimal to slight mononuclear inflammation was found in seven animals in group 3 and group 4. Slight BM thickening, a minimal focal to multifocal protein cylinder as well as a minimal to slight vacuolization was observed in most animals of all groups. Regeneration was solely found in six animals of group 4 and one animal of group 3. Additionally, spontaneous and background lesions, as for example pelvis dilatation, small cysts and minimal focal hemosiderosis, occurred occasionally. According to the renal damage score criteria of Rolleman et al., group 1 has the lowest average score (RDS 2.94), followed by group 3 (3.19), group 2 (3.25) and group 4 (highest score, 3.31). The obtained grading values were used to calculate means for all four groups and 95% confidence intervals, which are displayed in Table 5. The lowest average renal damage score was found in the group receiving placebo only, higher damage scores in groups 2 to 4. However, Pearson’s chi-squared test showed no significant difference between groups (*p* = 0.395).

**Table 5:** Renal damage score (RDS) expressed as means and 95% confidence interval

Treatment	RDS
Placebo	2.94 ± 0.15
Everolimus	3.25 ± 0.39
Placebo + [^177^Lu]Lu-DOTA-TATE	3.19 ± 0.32
Everolimus + [^177^Lu]Lu-DOTA-TATE	3.31 ± 0.39

## Discussion

The range of therapeutic options in advanced or metastatic NET is limited. If possible, metastasis resection or ablative techniques are used. For patients inappropriate for the aforementioned strategies, medical options can be somatostatin analogues, interferon-α, chemotherapy, Sunitinib or everolimus [20, 21]. In the RADIANT-3 trial, the median progression-free survival of patients treated with everolimus was 11.0 months compared to 4.6 months under placebo treatment. [^177^Lu]Lu-DOTA-TATE plus standard dose octreotide LAR has shown to be effective in midgut NETs [6]. As objective response rates are low (5% for everolimus in p-NETs, 18% for [^177^Lu]Lu-DOTA-TATE + octreotide LAR in midgut NETs) [6, 22], there is still need for optimizing therapeutic strategies, for example by combining established therapies. There has been some effort to combine other targeted agents with everolimus, but studies show either inacceptable toxicities [23] or only moderate clinical activity when using the maximum tolerated doses [24]. As already mentioned, the combination of everolimus and PRRT seems theoretically reasonable, however, due to the proposed synergistic effect and the dissatisfactory results of other combination studies, a combined therapy with everolimus and PRRT can only be used with particular caution. This is the first preclinical study to investigate the potentially aggravated toxicity of a combined treatment with everolimus and [^177^Lu]Lu-DOTA-TATE.

Rats receiving everolimus showed a slower weight gain than rats receiving placebo regardless whether it was combined with PRRT or not. This coincides with findings reported by Ramadan et al., who investigated the effects of everolimus on proteinuria in rats [25], which is consistent with the characteristics of everolimus as an inhibitor of cellular proliferation. Nevertheless, since weight gain based on growth processes plays a minor role in treatments with adult patients, this fact should be of minor importance in clinical practice. The altered levels of RBC, WBC and platelet count are not entirely unexpected, since everolimus is not only a cytoreductive but also an immunosuppressive agent and therefore partially modifies bone marrow activity. However, despite reaching statistical significance in our analysis, the changes are very moderate. These findings, as well as the rise in neutrophil counts and the equality of the monocyte counts after administration of everolimus, are in line with observations by Chen et al., who monitored haematological parameters in patients with metastatic breast cancer treated with everolimus [26]. The mechanism of everolimus causing these changes remains unclear. Rolleman et al. showed that PRRT with [^177^Lu]Lu-DOTA-TATE compromises haemoglobin levels in rats in a dose-dependent manner [19]. In our study, we used a slightly lower dose of [^177^Lu]Lu-DOTA-TATE, which resulted in a non-significant decline in serum haemoglobin. This indicates that our dose was selected reasonably, and haematotoxicity is increasing measurably when combining PRRT with everolimus. However, due to the effects of both therapies on these parameters, it is difficult to interpret the RBC, haemoglobin and haematocrit levels regarding the haematotoxicity in a combined regime. Both therapies reduce platelet and leucocyte counts. The group receiving the combined therapy showed the lowest group means for these parameters. In this context, the reduction of WBC was shown to be significant when everolimus was applied. These findings indicate that the impairment of both cellular immunity and platelet count might be a relevant issue for future studies on the combination of everolimus and [^177^Lu]Lu-DOTA-TATE, particularly, as it is difficult to protect the bone marrow from radiation.

In terms of renal damage in the patient setting, kidneys are protected by administrating amino acids, which prevents severe adverse events as shown in the NETTER-1 trial [6]. However, in the present work, no amino acids or other nephroprotective agents were applied to protect the kidneys in order to be able to detect differences in the extent of renal function impairment. Laboratory analysis showed that creatinine levels increase particularly in the group receiving PRRT and placebo. The increase of creatinine levels is significantly lower in rats receiving everolimus, which is in line with better excretion revealed by the later and higher peak in the renograms. This fact may be an indication of nephroprotective characteristics of everolimus in animals treated with PRRT and might be explained by the fact that everolimus can inhibit the expression of the megalin receptor as reported by Gleixner et al. [27], which will reduce the re-uptake of [^177^Lu]Lu-DOTA-TATE in the proximal tubules. Furthermore, Ramadan et al. confirmed that toxic effects of Adriamycin in rats can be mitigated significantly when everolimus is applied [25]. Additionally, renal scintigraphies showed that the FUR of [^99m^Tc]Tc-MAG3 was significantly lower after 16 weeks (day 112) in groups 3 and 4, both receiving PRRT regardless whether everolimus was combined or not. Since [^99m^Tc]Tc-MAG3 is mainly excreted by the proximal tubules, this finding is in accordance with a selective impairment of proximal tubular function after treatment with [^177^Lu]Lu-DOTA-TATE. Considering the laboratory results for creatinine and BUN, the results of the scintigraphies and the histological analysis of the kidneys, a slight impairment of renal function is caused by [^177^Lu]Lu-DOTA-TATE, which does not result in significant differences in renal damage scores. Interestingly, in a study on renal toxicity of [^177^Lu]Lu-DOTA-TATE conducted by Rolleman et al., renal damage scores in untreated control animals were 0.5 on average, which is far below the average score of 2.94 found in rats treated with placebo in our study. Theoretically, a potential explanation of these findings could be a nephrotoxic effect of sequential renal scintigraphies. However, the fact that histological patterns of renal damage were also present animals that were used for laboratory analysis only contradicts this hypothesis. However, repeated application of inhalational anaesthesia with isoflurane, which was used for blood sampling and scintigraphies, could be nephrotoxic by inducing hypotension and, therefore, reducing renal blood flow. Measurements with an additional group of animals without any anaesthesia might be reasonable to verify this hypothesis; however, this was not performed due to restrictions by our institutional guidelines and the Administrative Panel on Laboratory Animal Care of Upper Bavaria. The average RDS, however, is not further compromised in rats treated with everolimus and/or [^177^Lu]Lu-DOTA-TATE. This is in line with another finding by Rolleman et al. [18]. When analysing the long-term toxicity of the treatment with [^177^Lu]Lu-DOTA-TATE in rats, no correlation of morphological renal damage and rise in creatinine levels was observed even after application of higher cumulative doses of PRRT. As hypothesized by Rolleman et al., the reason can be a potentially very inhomogeneous functional reserve in the severely damaged kidneys. This effect may also apply for this study, as all the kidneys seem to be strongly affected according to morphological criteria.

In summary, it is to be assumed that renal scintigraphies using [^99m^Tc]Tc-MAG3 show high sensitivity for the detection of even slight changes of renal function. Nonetheless, our data do not indicate an increased renal or haematological toxicity by a combined treatment with everolimus and [^177^Lu]Lu-DOTA-TATE compared to the mere treatment with [^177^Lu]Lu-DOTA-TATE alone.

## Conclusion

Our preclinical data on the combined toxicity of [^177^Lu]Lu-DOTA-TATE and everolimus do not show increased toxicities compared to the monotherapies. Thus, further evaluation of the efficacy of a combined therapy using everolimus and [^177^Lu]Lu-DOTA-TATE in tumour bearing animals is highly feasible. Potential synergistic anti-tumour effects on AR42J tumour bearing rodents are currently performed at our institution.

## Data Availability

The datasets generated during and/or analysed during the current study are available from the corresponding author on reasonable request.

## Abbreviations

ANOVA: Analysis of variance
BUN: Blood urea nitrogen
DOTA: 1,4,7,10-Tetraazacyclododecane-1,4,7,10-tetraacetic acid
DOTA-TATE: DOTA^0^,Tyr^3^-octreotate
DOTA-TOC: DOTA^0^,Tyr^3^-octreotide
FUR: Fractional uptake rate
HE: Haematoxylin-eosin
LU: Linear uptake
MAG3: Mercaptoacetyltriglycine
mTOR: Mechanistic target of rapamycin
NET: Neuroendocrine tumour
PAS: Periodic acid-Schiff
PR: Patlak-Rutland
PRRT: Peptide receptor radionuclide therapy
RBC: Red blood cell
RDS: Renal damage score
ROI: Region of interest
SRH: Scheirer-Ray-Hare
WBC: White blood cell
